# New method of clustering colorectal cancer patients using differential presence of exons (DPE) sequencing

**DOI:** 10.18632/oncoscience.573

**Published:** 2023-03-23

**Authors:** David Rubio-Mangas, Mariano García-Arranz, Javier Suela, Damian García-Olmo

**Keywords:** whole-exome sequencing, NGS, cell-free DNA, DPE, differential presence of exons

Colorectal cancer (CRC) is a heterogeneous disease that occurs in the colon and the rectum, parts of the gastrointestinal system [1]. CRC is the third leading cause of cancer-related death worldwide [2]. The incidence and mortality of CRC is expected to increase significantly in the future, with more than 2.2 million new cases and 1.1 million deaths expected by 2030 [3]. Metastasis is the leading cause of death in CRC patients, especially liver metastasis. According to previous studies, about 25% of CRC cases are clinically diagnosed with liver metastases in early stages, and about 50% of CRC patients experience symptoms of liver metastases throughout the course of the disease [4–6].

Differential presence of exons (DPE) by next-generation sequencing (NGS) is an innovative method to analyze the complete exome sequence and can be used as a stratification and predictive tool in patients with colorectal cancer (CRC) [7, 8]. CRC is one of the most common neoplasms worldwide, and often presents at advanced stages, making it difficult to treat and decreasing survival rates [9]. Early detection of CRC and its stratification is crucial to improve disease prognosis and reduce mortality [10].

**Figure 1:**
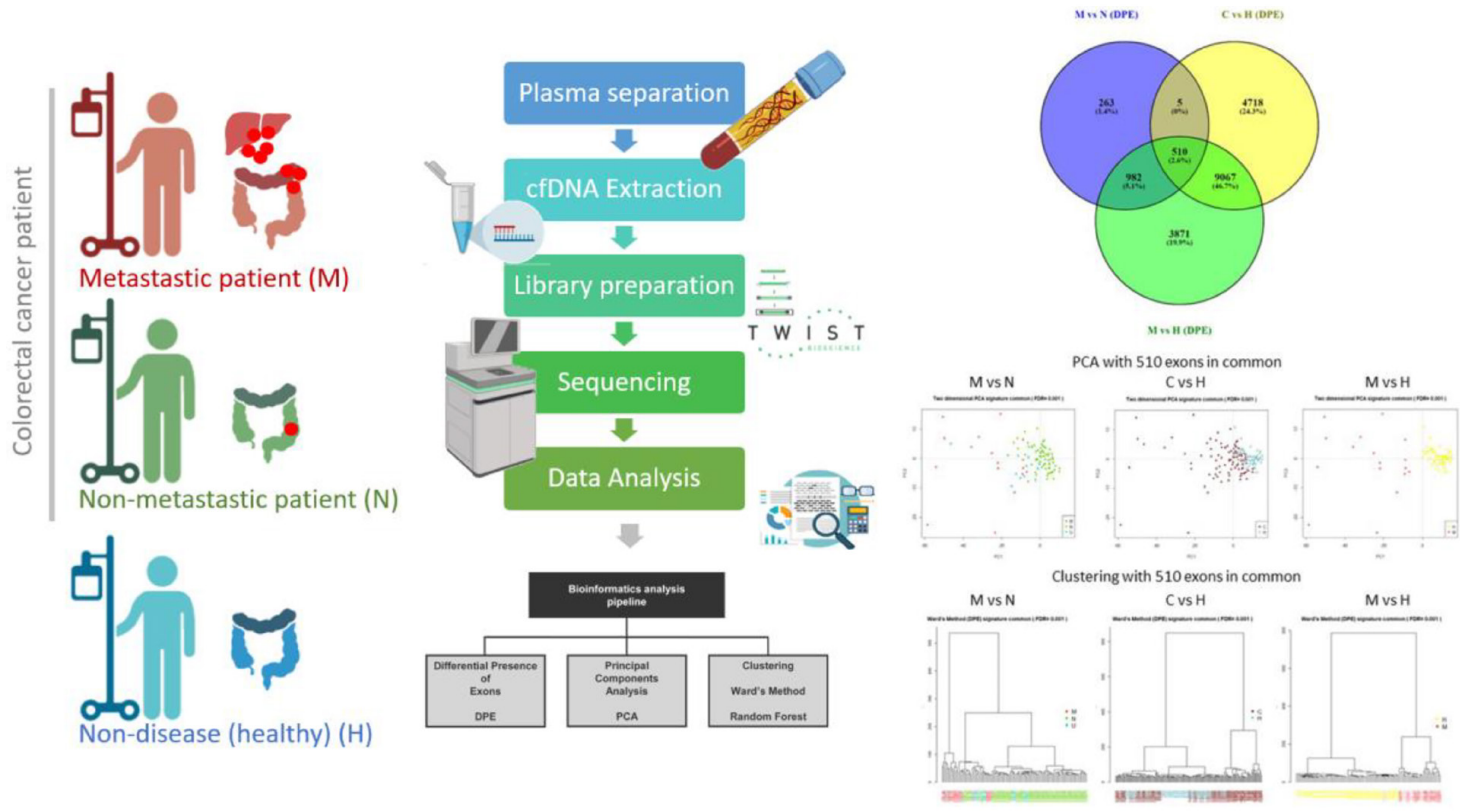
Schematic workflow of the experimental procedure. Cell-free DNA (cfDNA) was isolated from plasma of patients with colorectal cancer (CRC) and healthy donors. Exome capture was performed before sequencing, and the resultant reads were subsequently aligned to the reference genome sequence (hg38). A pipeline for NGS data analysis was applied to cfDNA from patients with CRC and healthy donors. Exploratory analysis of common exons in the three groups of patients studied. A Venn diagram of all comparisons using the Venny platform. As we can observe, there are 510 exons in common from the three comparatives (M vs. N, C vs. H and M vs. H) and these exons were used an exonic signature related to colorectal cancer. Two-dimensional principal component analysis (PCA) plot with the 510 exons in common (exonic signature). Using samples from the comparative M vs. N, C vs. H and M vs. H. It can be seen that there is discrimination between the groups using these 510 exons. Samples from groups M, N, U and H are marked with different colors according to the graph. The graph on the left: M in red, N in green, and U in blue. The middle graph: C in brown and H in blue. The graph on the right: M in red and H in yellow. C Clustering of the samples by Ward’s method with the 510 exons in common (exonic signature). We can see the clustering on the left: M in red, U in blue and N in green. It is clear that there is separation between the clusters. In the middle cluster: C in brown and H in blue, the separation between groups is also observed and in the cluster on the right: M in red, U in pink and H in yellow, the separation between groups is also observed. Unclassifiable patients (U) were placed in the center, indicating that they share features with both groups. Abbreviations: M: Metastatic; N: Non-metastatic; C: Colorectal cancer patients; H: healthy controls. U; patients with indeterminate colorectal cancer (metastasic and non-metastatic).

In a recent study, a common exonic signature of 510 exons was identified whose differential presence in plasma allowed us to discriminate and classify between different clinical pictures, including patients with metastatic CRC, patients with non-metastatic CRC and healthy individuals [8]. Using a novel and sustainable technology in liquid biopsy, the DPE analysis revealed significant differences in the levels of DNA exons circulating in the plasma of CRC patients compared to healthy individuals, as well as metastatic CRC patients compared to non-metastatic and metastatic compared to healthy individuals (Figure 1). Furthermore, this analysis yielded highly significant values, suggesting that circulating DNA in the patient’s plasma may be involved in intercellular communication and may play a key role in malignant transformation (genometastasis) [11].

DPE analysis has emerged as a novel and promising technique in the field of liquid biopsy and has the advantage of being non-invasive compared to invasive diagnostic techniques. In addition, this analysis can provide valuable information on CRC progression and could provide information on response to therapy. The use of liquid biopsy in clinical practice may also reduce the costs and time required for staging CRC patients, which could have implications for diagnosis and treatment [12, 13].

DPE analysis may also be useful in identifying new biomarkers that can predict CRC progression. The identification of specific biomarkers may allow the identification of patients with metastatic CRC and the selection of personalized and more effective therapies.

In conclusion, DPE analysis may be a promising tool in the identification of metastatic CRC and in the classification of different clinical pictures. The results of this study may have important implications for the development of personalized and more effective therapies for CRC patients. The use of liquid biopsy and DPE analysis in clinical practice may improve the efficacy of CRC diagnosis and treatment, which may lead to longer survival and improved quality of life for patients. Further studies are needed to validate these findings and to validate the clinical utility of DPE analysis in the stratification of CRC patients. Nevertheless, the study is promising and suggests that DPE analysis may have an important role in improving the diagnosis and management of CRC.
